# Review of Huang-huai sheep, a new multiparous mutton sheep breed first identified in China

**DOI:** 10.1007/s11250-020-02453-w

**Published:** 2020-11-23

**Authors:** Kai Quan, Jun Li, Haoyuan Han, Hongfang Wei, Jinyan Zhao, HA Si, Xinji Zhang, Daojiang Zhang

**Affiliations:** 1 Henan University of Animal Husbandry and Economy, Zhengzhou, 450046 Henan China; 2 Lvyuan Mutton Sheep Development Co., Ltd., Wuyang, 462400 Henan China; 3 Xunxian Xinlin Animal Husbandry Co. Ltd., Xunxian, 456282 Henan China

**Keywords:** Huang-huai sheep, Hereditary, Reproduction, Production, Adaptability

## Abstract

Huang-huai sheep are a new multiparous mutton sheep breed that has been cultivated by domestic scientific research institutes, governments, and sheep farms in China. Huang-huai sheep were bred using Dorper sheep as a sire and Small-tailed Han sheep as a dam. The breeding of Huang-huai sheep started in 2003, and three stages have been carried out: crossbreeding innovation, fixation in a two-way-crossbred closed flock, and herd propagation. A pilot test of Huang-huai sheep was conducted on 6 sheep farms from 2017 to 2018, and hereditary properties and production performance were evaluated in 2019. Huang-huai sheep were identified on site by the National Livestock and Poultry Resources Committee of China in December 2019 and approved as a new multiparous mutton sheep breed in China. The genetic distance showed that Huang-huai sheep are most closely related to Dorper sheep, Luxi black-headed sheep, and Small-tailed Han sheep, but the genetic distances are subspecies (0.02–0.20) each other. The body weights of adult Huang-huai sheep are 98.1 ± 5.2 kg (♂) and 71.7 ± 3.5 kg (♀), and those of 6-month-old Huang-huai sheep are 58.50 ± 6.55 kg (♂) and 52.45 ± 5.67 kg (♀). The slaughter rates of 6-month-old sheep are 56.02 ± 1.25% (♂) and 53.19 ± 1.19% (♀). The estrus cycle of Huang-huai sheep is 19.32 ± 2.8 days, the first estrus cycle occurs at 168 ± 12 days, the annual lambing rate of ewes is 252.82% ± 10.69%, the survival rate of lambs is 95.79 ± 0.95%, and the number of weaned lambs per ewe per year is 2.38 ± 0.14. The growth performance, carcass quality, and reproductive performance of Huang-huai sheep have been improved, resulting in considerable economic and social benefits and broader market prospects. This breed represents a new multiparous mutton sheep breed adapted for industrial sheep farms in China.

## Introduction

An excellent breed of livestock is the foundation for animal husbandry (Abraham et al. 2018). The Central China Plain area is located in the warm temperate zone and has a humid climate, ample sunshine, and convenient irrigation conditions. It has been recognized as an important grain-producing area in China (Tie et al. 2016). The climate and natural resources of the Central China Plain are very suitable for industrial agriculture and animal husbandry, especially mutton sheep farming (Li et al. 2020). Multiparity in mutton sheep has become crucial for mutton sheep farming in the Central China Plain, and thus, it is important to breed a new multiparous mutton sheep breed suitable for the Central China Plain (Liu et al. 2016). With economic development, mutton consumption is increasing (Zeng et al. 2019). Therefore, industrialized sheep farming is needed in China. Industrial sheep farming is necessary for fast growth, a high carcass ratio and reproductive rate, adaptability to the environment, and suitability for house raising (Montossi et al. 2013). However, there are no such sheep breeds in the Central China Plain area.

To breed an industrialized mutton sheep, we used Dorper sheep as a sire and Small-tailed Han sheep as a dam to breed a new multiparous mutton sheep breed adapted to the Central China Plain that meets the needs for industrialized mutton in China. Dorper sheep were introduced from Australia to Henan Province in 2003 and have the advantages of fast growth, a high carcass ratio, and high meat quality, but the reproductive rate is only 120% (Cloete et al. 2000). Small-tailed Han sheep are a prolific local sheep breed in China and have the advantages of good local adaptability, a high reproductive rate, and an average litter size of 2 (Yuan et al. 2019), but the mutton performance and coarse feeding resistance are relatively poor. Huang-huai sheep combine the advantages of Dorper and Small-tailed Han sheep.

Huang-huai sheep are mainly distributed in the central and eastern regions of Henan Province, the northern region of Anhui Province, and the northwest region of Jiangsu Province. The distribution of this breed covers the entire Central China Plain, which is the golden belt for sheep breeding and the national core area of the sheep and grain industries. The Central China Plain has abundant straw resources and unique conditions suitable for the sheep industry. In Henan Province, 21.45 million sheep were economically fattened, and 16.82 million were stocked in 2017. The annual output value of mutton sheep was 40 billion yuan, and the total purchase of breeding sheep exceeded 4 billion yuan. The purchase of breeding sheep from Henan Province accounts for less than 10% of the total purchase, but the purchase of introduced breeding sheep exceeds 400 million yuan each year. More than 80% of breeding sheep are introduced. The number of people who engage in the mutton sheep industry is more than 1.5 million in Henan Province, and most of these people have intermediate to low incomes. An increase in mutton performance of 10% would lead to an increase of more than 3 billion yuan for the new mutton sheep breed and more than 2000 yuan per person.

## Materials and methods

### Breeding materials

A total of 52 Dorper rams from 6 Dorper sheep farms belonging to 19 lineages were used as sires (Table 1), and 8400 Small-tailed Han ewes from 8 breeding farms were used as dams (Table 2).

**Table 1:** Source sheep farms and lineage of rams

Source sheep farms of ram	Number of rams (*n*)	Lineages (*n*)
Henan Dorper Industrial Co., Ltd.	25	6
Dongying Chaoqun Livestock Co., Ltd.	5	2
Tianjin Aoqun Animal Husbandry Co., Ltd.	5	2
Ningxia Nongxia Luning Small Tail Han Sheep Breeding Center (Co., Ltd.)	7	3
Siziwangqi Saino Animal Husbandry Technology Co., Ltd.	5	3
Shandong Kaiyin Livestock Technology Development Co., Ltd.	5	3
Total	52	19

**Table 2:** Huang-huai sheep core breeding ground

Breeding farm	Number of ewes (*n*)	Address
Xunxian Xinlin Animal Husbandry Co., Ltd.	1000	Xun County, Henan Province
Henan Lvyuan Meat Sheep Development Co., Ltd.	1000	Wuyang County, Henan Province
Henan Dorper Industrial Co., Ltd.	800	Zhongmou County, Henan Province
Yuzhou Jinhaolong Animal Husbandry Co., Ltd.	1000	Yuzhou City County, Henan Province
Henan Yihao Agricultural Technology Co., Ltd.	1000	Song County, Henan Province
Henan Sanmu Lvyuan Animal Husbandry Co., Ltd.	2000	Dengfeng County, Henan Province
Qixian Yonghe Agriculture & Animal Husbandry Co., Ltd.	800	Qi County, Henan Province
Fengqiu Yingju Circular Agriculture Co., Ltd.	800	Fengqiu County, Henan Province
Total	8400	

### Breeding technique and course

**Fig. 1:**
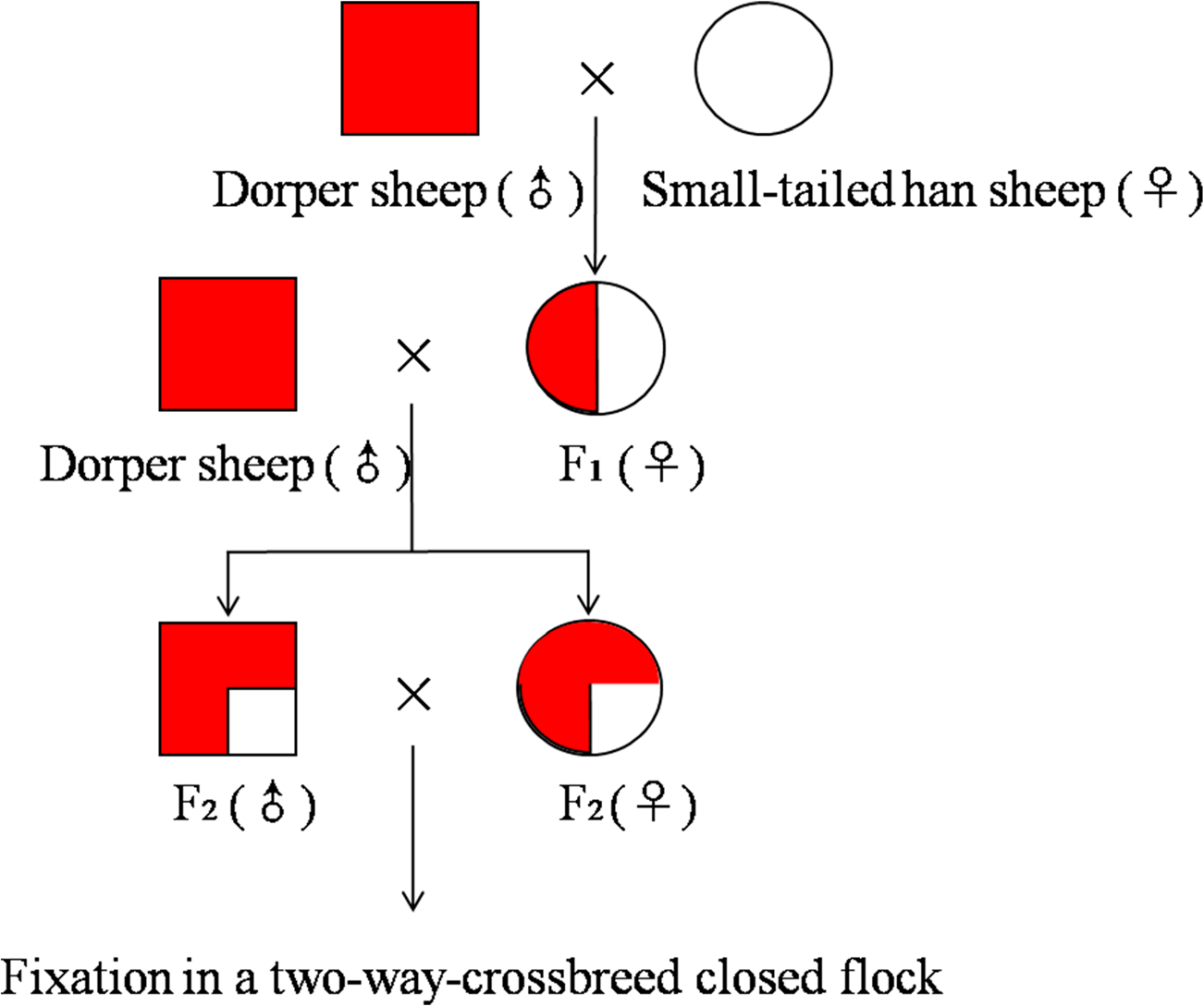
Crossbreeding innovation. The second-generation (F_2_) hybrid flocks produced from Dorper sheep and Small-tailed Han sheep contained 75% Dorper sheep genetic material and 25% Small-tailed Han sheep genetic material

The traditional binary crossbreeding method was used, and the breeding process was divided into three stages: crossbreeding innovation (Fig. 1), fixation in a two-way-crossbreed closed flock, and herd propagation. Crossbreeding innovation began in 2003, in which Dorper sheep were introduced and hybridized with local Small-tailed Han sheep at 8 breeding farms in Henan Province. Dorper rams were adopted for gradation hybridization in 2008. Fixation in a two-way-crossbred closed flock was carried out beginning in 2012, and modern advanced breeding technologies such as open core breeding and marker-assisted selection were adopted. Herd propagation began in 2018.

### Pilot test

The pilot test of Huang-huai sheep was conducted with 12,000 sheep from 6 sheep farms from 2017 to 2018 (Table 3). The genetic stability, live weight, conformation, carcass traits, reproductive traits, and economic value of Huang-huai sheep were evaluated according to the “Measures for Administration of Pilot Test of Breeding Livestock and Poultry” of the *Ministry of Agriculture and Rural Affairs* of China.

**Table 3:** Pilot test farms of Huang-huai sheep

Pilot test farms	Number (*n*)	Address
Anhui Huangzhu Animal Husbandry Technology Co., Ltd.	1000	Dingyuan County, Anhui Province
Anhui Qingqingcao Animal Husbandry Technology Development Co., Ltd.	1000	Yingshang County, Anhui Province
Luoning Sanyang Animal Husbandry Co., Ltd.	3000	Luoning County, Henan Province
Neixiang Hongmu Breed Sheep Co., Ltd.	2000	Neixiang County, Henan Province
Henan Sanyang Livestock Co., Ltd.	3000	Changyuan County, Henan Province
Henan Kunyuan Agriculture and Animal Husbandry Technology Co., Ltd.	2000	Ruzhou County, Henan Province
Total	12,000	

### Genetic testing

Genetic testing was conducted in the Biological Laboratory of Henan University of Animal Husbandry and Economics from December 2018 to January 2019. A total of 50 blood samples, including samples from 20 Huang-huai sheep, 10 Small-tailed Han sheep, 10 Dorper sheep, and 10 Luxi black-headed sheep, were collected. Genomic DNA was extracted with a DNA extraction kit (Aidlab Biotechnologies Co., Ltd., Beijing, China), and the PCR primers for the mutton D-loop sequence (accession number: AF039578) were F: 5′-AGCCCCACTATCAACACC-3′ and R: 5′-AAATAGTTACCCCCACAGTTAG-3′. PCR was performed in a 20-μL reaction containing 10 μL of Taq PCR Master Mix (1 U Taq Polymerase, 5 μmol L^−1^ dNTP, 0.2 mM Tris-HCl, 1 mmol L^−1^ KCl, 0.03 mmol L^−1^ MgCl_2_), 1 μL of each primer (10 μmol L^−1^), 1 μL of pooled genomic DNA (100 ng L^−1^), and 7 μL of ddH_2_O. Template DNA was initially denatured at 95 °C for 3 min, followed by 34 cycles of PCR amplification with the following conditions: 30 s at 95 °C, 35 s at 54 °C, 45 s at 72 °C, a final extension for 5 min at 72 °C, and finally stored at 4 °C. The amplified products were sequenced by an ABI PRIZM 377 DNA sequencer (Perkin-Elmer) (Shanghai Sangon Biotech Company, Shanghai, China). The sequencing results were aligned by MEGA 6.0, and a neighbor-joining phylogenetic tree was constructed. The genetic distance between breeds was measured by the Kimura 2-parameter model. Haplotype diversity, nucleotide diversity, genetic differentiation, the average number of nucleotide differences (Kxy), and nucleotide divergence (Dxy) were analyzed by DnaSP V5.

### Performance test

Body weight and body size were measured in 120 Huang-huai sheep by the *Department of Agriculture-Breeding Sheep and Wool Quality Supervision Test Center* of the Ministry of Agriculture and Rural Affairs of China (Hohhot). The data were analyzed by ANOVAs and Duncan’s test with SPSS 22.0.

### On-site validation

Huang-huai sheep were identified at the two core breeding farms (Xinlin Animal Husbandry Co., Ltd., and Lvyuan Mutton Sheep Development Co., Ltd.). The live weight and body size of 120 Huang-huai sheep were examined, and the breeding process and materials were audited by the *Sheep Resources Committee of the National Livestock and Poultry Breeding Committee of China* in December 2019.

## Results

### Hereditary properties

**Fig. 2:**
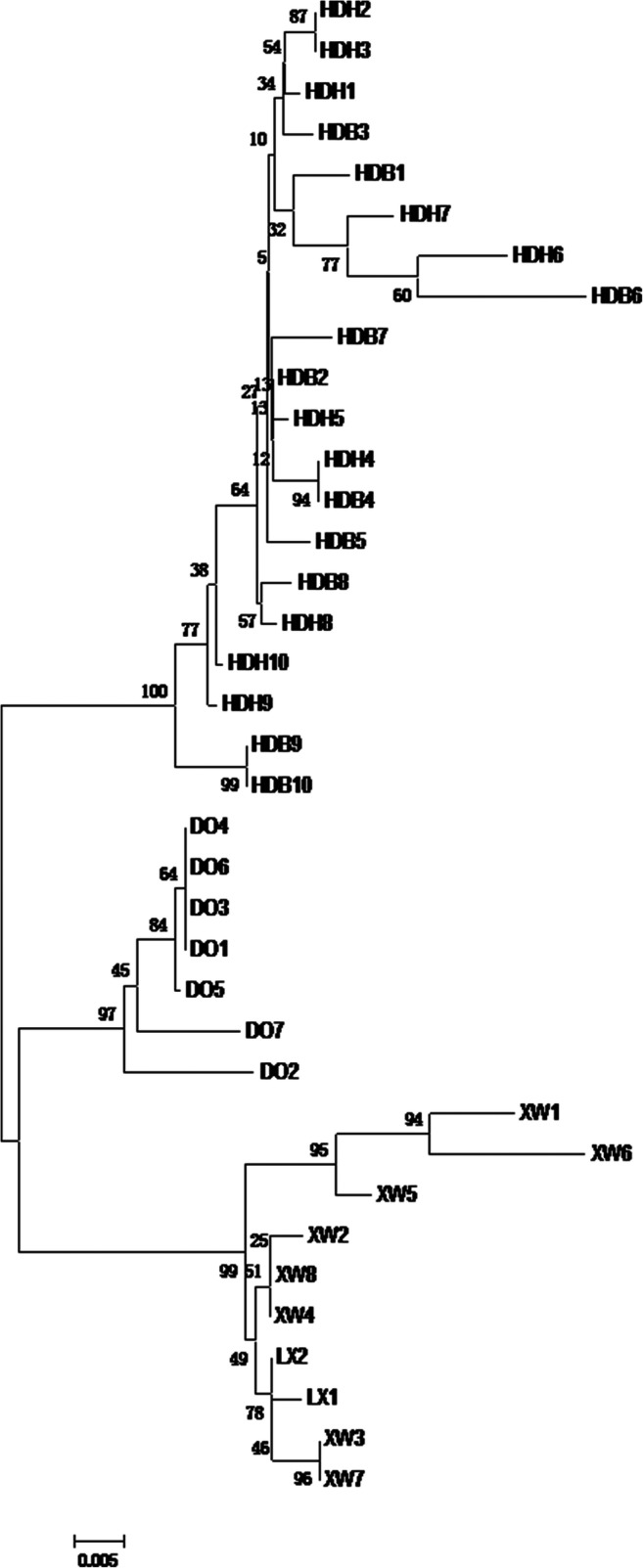
Phylogenetic trees of four sheep breeds. DO is Dorper sheep, HD is Huang-huai sheep, LX is Luxi black-headed sheep, and XW is Small-tailed Han sheep

Based on the transition and transversion of D-loop sequences, a neighbor-joining phylogenetic tree of Huang-huai sheep, Small-tailed Han sheep, Dorper sheep, and Luxi black-headed sheep was constructed. Huang-huai sheep clustered together, and Luxi black-headed sheep and Small-tailed Han sheep clustered together first and then clustered with Dorper sheep (Fig. 2).

Genetic distances were measured by MEGA 6.0 based on the Kimura 2-parameter model. The genetic distance of Huang-huai sheep was 0.055 from Dorper sheep, 0.063 from Luxi black-headed sheep, and 0.070 from Small-tailed Han sheep (Table 4), all of which were consistent with genetic distances between subspecies (0.02–0.20). Therefore, Huang-huai sheep are most closely related to Dorper sheep, Luxi black-headed sheep, and Small-tailed Han sheep.

**Table 4:** Genetic distance between breeds

	Huang-huai sheep	Dorper sheep	Luxi black-headed sheep	Small-tailed Han sheep
Huang-huai sheep	–			
Dorper sheep	0.055	–		
Luxi black-headed sheep	0.063	0.070	–	
Small-tailed Han sheep	0.070	0.053	0.016	–

### Phenotypic characteristics

**Fig. 3:**
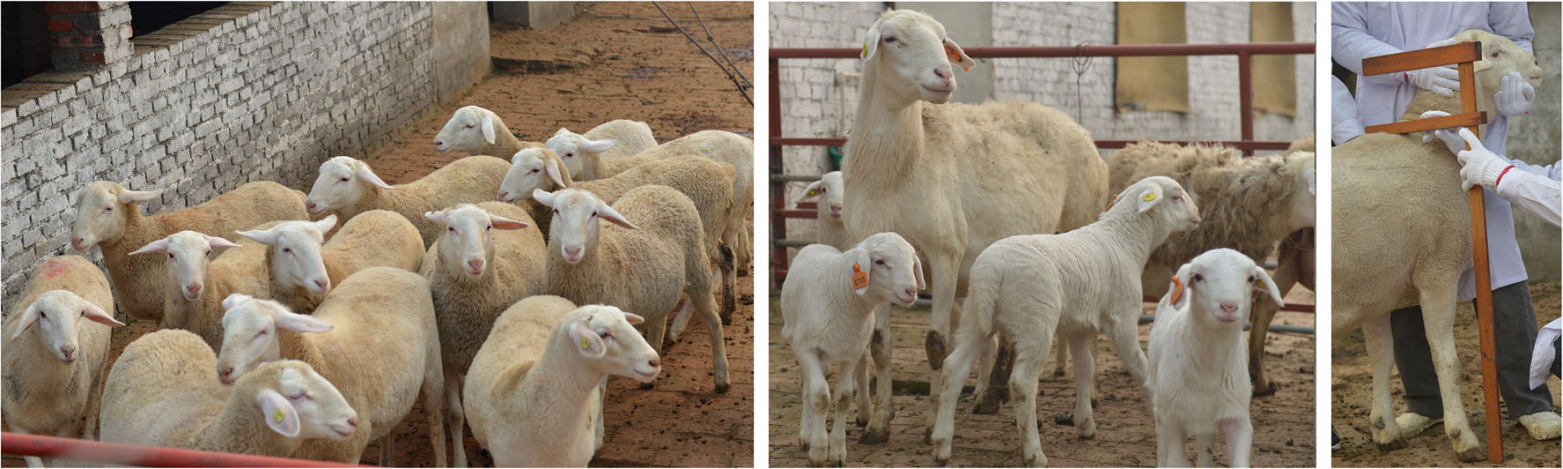
Images of Huang-huai sheep. **a** Young ewes flock, **b** ewes and lambs, and **c** measuring the ram body size

There are two groups of Huang-huai sheep: sheep with a black head and sheep with a white head. Black-headed sheep have black hair and skin from head to neck, white hair and skin on the body, and black hair and skin around the anus and vulva. The coat and skin of the white-headed Huang-huai sheep are white over the whole body, without variegation. Huang-huai sheep has an attractive face with an uplifted nose bridge, medium-sized, slightly droopy ears, and a good head and neck combination. Huang-huai rams have a stubby neck, and ewes have a slightly slender neck. The chest is wide and deep, the back and waist are flat and straight, and the hindquarters are plump, showing a doubly muscular rump. The four limbs are long and strong, the hoof is solid, the bodily form is round and barrel-shaped, and the rams and ewes have a thin tail and no horns (Fig. 3).

### Growth performance

The live weight and body size were measured (Table 5). The adult Huang-huai sheep were 98.1 ± 5.2 kg (♂) and 71.7 ± 3.5 kg (♀) in weight, and the 6-month-old Huang-huai sheep were 58.50 ± 6.55 kg (♂) and 52.45 ± 5.67 kg (♀) in weight.

**Table 5:** Huang-huai sheep live weight and body size

Index Age	Sex	Weight (kg)	Body height (cm)	Body length (cm)	Bust (cm)	Leg hip (cm)	Tube girth (cm)
Newborn	♂	3.68 ± 1.31	–	–	–	–	–
	♀	3.65 ± 1.26	–	–	–	–	–
45 days old	♂	17.30 ± 2.27	–	–	–	–	–
	♀	16.34 ± 1.31	–	–	–	–	–
6 months old	♂	58.50 ± 6.55	71.62 ± 3.42	85.32 ± 5.31	99.42 ± 7.41	67.24 ± 3.12	9.56 ± 1.24
	♀	52.45 ± 5.67	66.45 ± 3.85	75.64 ± 3.17	94.12 ± 7.56	64.61 ± 3.28	8.60 ± 0.88
1 year old	♂	79.43 ± 4.48	76.31 ± 3.24	89.79 ± 3.66	103.41 ± 7.65	68.58 ± 5.61	10.58 ± 0.98
	♀	65.12 ± 6.77	72.92 ± 3.64	80.75 ± 3.43	96.39 ± 7.74	61.92 ± 4.62	9.12 ± 0.75
Adult	♂	98.10 ± 5.23	79.07 ± 2.86	88.60 ± 2.34	109.01 ± 4.23	69.14 ± 4.65	11.54 ± 0.65
	♀	71.70 ± 3.50	75.62 ± 2.63	81.81 ± 2.57	103.85 ± 4.46	59.63 ± 4.71	10.22 ± 0.63

### Slaughter performance

The slaughter performance of 6-month-old Huang-huai sheep was tested (Table 6). The slaughter rates of 6-month-old sheep were 56.02 ± 1.25% (♂) and 53.19 ± 1.19% (♀).

**Table 6:** The slaughter performance of 6-month-old Huang-huai sheep

Test object	Sex	Quantity	Carcass weight (kg)	Net meat weight (kg)	Eye muscle area (cm^2^)	Meat to bone ratio	Slaughter rate (%)	Carcass net meat rate (%)
Huang-huai sheep	♂	6	32.94 ± 1.56^a^	27.01 ± 1.28^a^	24.50 ± 2.08^a^	4.56 ± 0.23^a^	56.02 ± 1.25^a^	82.01 ± 1.45^a^
	♀	6	27.86 ± 1.87^b^	22.71 ± 1.52^b^	21.24 ± 1.56^b^	4.42 ± 0.21^a^	53.19 ± 1.19^b^	81.53 ± 1.27^a^
Small-tailed Han sheep	♂	6	27.26 ± 2.03^b^	21.25 ± 1.75^bc^	18.69 ± 2.57^c^	3.54 ± 0.38^b^	51.08 ± 1.21^c^	77.95 ± 1.21^b^
Dorper and Hu sheep hybrid (F_1_)	♂	6	24.32 ± 1.78^c^	19.92 ± 1.84^c^	20.36 ± 2.19^bc^	4.53 ± 0.26^a^	54.5 ± 1.35^b^	81.91 ± 1.33^a^

Different letters on the shoulder of the same column indicate significant differences (*P* < 0.05), and no letters or the same letters indicate no significant differences (*P* > 0.05)

### Reproductive performance

The reproductive performance of Huang-huai ewes was estimated. The first heat of Huang-huai sheep occurred at 5 to 6 months of age, sexual maturity occurred at 6 to 7 months of age, the estrus cycle lasted for 19.3 ± 2.8 days, estrus duration was 33 to 46 h, and the annual lambing rate of ewes was 252.82% ± 10.69%. Huang-huai sheep remain in estrus year round and can have 3 offspring in 2 years.

### Adaptability

The survival rate of lambs was 95.79 ± 0.95%, and the number of weaned lambs per ewe per year was 2.38 ± 0.14 (Table 7). This shows that Huang-huai sheep meet the specialized breed standards for industrialized development in the Central China Plain.

**Table 7:** Number of lambs produced by Huang-huai sheep

Item		Number of lambs (*n*)	Lamb production rate (%)	Total number of lambs (*n*)	Average number of lambs (*n*)	Average number of litters (*n*)	Annual reproductive rate (%)
First-born ewe	Single	403	62.77	883	1.38	1.76	252.82
	Double	237	36.92				
	Three	2	0.31				
Given-birth ewe	Single	404	23.99	3109	1.85		
	Double	1139	67.64				
	Three	141	8.37				
	Four	1	0.06				

The average litter size = the number of lambs born in the first womb × 20% + the number of lambs born in labor × 80% = 1.38 × 20% + 1.85 × 80% = 1.76 (*n*); annual reproduction rate (100%) = average the number of litters in the litter × annual breeding cycle = 1.76 × 143.65 = 252.82%

## Discussion

### Hereditary properties

The phylogenetic tree showed that Huang-huai sheep are genetically distinct from Luxi black-headed sheep, Dorper sheep, and Small-tailed Han sheep. The genetic distances between Huang-huai sheep and Dorper sheep, Luxi black-headed sheep, and Small-tailed Han sheep were 0.055, 0.063, and 0.070, respectively, all of which were consistent with genetic distances between subspecies (Starič et al. 2020). During the process of Huang-huai sheep breeding, the lineage of Dorper sheep contributed 75.00% of the genetic material, and that of Small-tailed Han sheep contributed 25.00%. After more than four generations of crossbreeding, Huang-huai sheep were most closely related to Dorper sheep. The black- and white-headed Dorper sheep acted as the paternal line for Huang-huai sheep, resulting in black- and white-headed Huang-huai sheep, which belong to one breed according to genetics (Cloete et al. 2000).

Our study proved that Huang-huai sheep are most closely related to Dorper sheep and least closely related to Small-tailed Han sheep. The Kxy and Dxy results corresponded to those observed for genetic distance. Huang-huai sheep showed abundant genetic diversity and a large effective population size, which reached the breeding targets for Huang-huai sheep.

### Growth performance

Weaning weight comprehensively reflects the lactation level of ewes, genetic performance, feeding and nursing levels of lambs, and adaptability (Signer-Hasler et al. 2019; Manzari et al. 2019). The weaning weight of Huang-huai lambs reached 17.30 kg (♂) and 16.34 kg (♀) at the 45th day, and strong maternal intuition in Huang-huai ewes was confirmed, which fit the standards for industrialized mutton sheep.

### Reproductive performance

The Dorper is a hardy South African composite breed derived from a cross between the black-headed Persian and the Dorset Horn, and the reproduction rate of Dorper ranges from 0.99 to 1.40 lambs per annum (Cloete et al. 2000). Small-tailed Han sheep are the most widely raised and famous maternal sheep breeds in China and are known for precocious puberty, perennial estrus, and high fecundity; the average litter size is over 2 (Yuan et al. 2019). The estrus of Huang-huai ewes occurred earlier than that of Dorper, Suffolk, Texel, and hornless Dorset ewes. The average litter size and the annual reproduction rate of Huang-huai sheep are much higher than those of Dorper, Texel, Suffolk, hornless Dorset, and Charolais sheep (Hernández et al. 2017). This shows that the reproductive performance of Huang-huai sheep meets the specialized breed standards for industrialized mutton sheep.

### Adaptability

The weaning survival rate and the number of weaned lambs per ewe of Huang-huai sheep are higher than those of Small-tailed Han sheep, Hu sheep, and Bamei mutton sheep (He et al. 2016; Ma et al. 2018). The number of weaned lambs provided by ewes in a year reflects not only the breed’s reproductive rate but also its environmental adaptability, the maternity of the breed, and the feeding and management levels of the farm. This shows that Huang-huai sheep meet the specialized breed standards for industrialized development in the Central China Plain.

### Slaughter performance

The slaughter percentage of 6-month-old Huang-huai sheep was 56.02%, the carcass weight was 32.94 kg, the net meat weight was 27.01 kg, and the eye muscle area was 24.50 cm^2^. These results showed that the performance of Huang-huai sheep accorded with and partly exceeded that of international and domestic specialized mutton sheep. Carcass weight is the ultimate representation of economic benefit. The eye muscle area, the main index used to evaluate high-end mutton, of Huang-huai sheep is somewhat high.

### Evaluation and outlook

Huang-huai sheep are easy to manage and adapt to the scale of feeding or breeding. Using conventional breeding and marker-assisted selection, we constructed a genetic evaluation system for Huang-huai sheep. This system integrates standardized large-scale breeding and an industrial development technology system for Huang-huai sheep, making it possible to combine large-scale livestock enterprises, breeding farms, and farmers. This will establish an industrial mode that develops the rural economy and drives farmers out of poverty by supporting livestock enterprises.

In conclusion, Huang-huai sheep have improved growth performance, carcass quality, reproductive performance, more considerable economic and social benefits, and broader market prospects than other breeds. This breed will provide strong support for the development of the modern mutton sheep industry, which is needed not only for the market but also for social development in China.

## Data Availability

I certify that all data in the article are true and reliable, and all data have been verified by Chinese government authorities (third-party testing data). This manuscript is original and has not been submitted elsewhere for publication.

## References

[CR1] Abraham H, Gizaw S, Urge M (2018). Identification of breeding objectives for Begait goat in western Tigray, North Ethiopia. Tropical Animal Health and Production.

[CR2] Cloete SW, Snyman MA, Herselman MJ (2000). Productive performance of Dorper sheep. Small Ruminant Research : the journal of the International Goat Association.

[CR3] He X, Zhou Z, Pu Y, Chen X, Ma Y, Jiang L (2016). Mapping the four-horned locus and testing the polled locus in three Chinese sheep breeds. Animal Genetics.

[CR4] Hernández JA, Lepe M, Macedo R, Arredondo V, Cortez CE, García LJ, Prado O (2017). Morphological study of Socorro Island Merino sheep and its crosses with hair breeds. Tropical Animal Health and Production.

[CR5] Li Q, Lu Z, Jin M, Fei X, Quan K, Liu Y, Ma L, Chu M, Wang H, Wei C (2020). Verification and Analysis of Sheep Tail Type-Associated PDGF-D Gene Polymorphisms. Animals : An Open Access Journal from MDPI.

[CR6] Liu Z, Ji Z, Wang G, Chao T, Hou L, Wang J (2016). Genome-wide analysis reveals signatures of selection for important traits in domestic sheep from different ecoregions. BMC Genomics.

[CR7] Ma L, Li Z, Cai Y, Xu H, Yang R, Lan X (2018). Genetic variants in fat-and short-tailed sheep from high-throughput RNA-sequencing data. Animal Genetics.

[CR8] Manzari Z, Mehrabani-Yeganeh H, Nejati-Javaremi A, Moradi MH, Gholizadeh M (2019). Detecting selection signatures in three Iranian sheep breeds. Animal Genetics.

[CR9] Montossi F, Font-i-Furnols M, del Campo M, San Julián R, Brito G, Sañudo C (2013). Sustainable sheep production and consumer preference trends: compatibilities, contradictions, and unresolved dilemmas. Meat Science.

[CR10] Signer-Hasler H, Burren A, Ammann P, Drögemüller C, Flury C (2019). Runs of homozygosity and signatures of selection: a comparison among eight local Swiss sheep breeds. Animal Genetics.

[CR11] Starič J, Farci F, Luridiana S, Mura MC, Pulinas L, Cosso G, Carcangiu V (2020). Reproductive performance in three Slovenian sheep breeds with different alleles for the MTNR1A gene. Animal Reproduction Science.

[CR12] Tie X, Huang RJ, Dai W, Cao J, Long X, Su X, Zhao S, Wang Q, Li G (2016). Effect of heavy haze and aerosol pollution on rice and wheat productions in China. Scientific Reports.

[CR13] Yuan Z, Zhang J, Li W, Wang W, Li F, Yue X (2019). Association of Polymorphisms in Candidate Genes with the Litter Size in Two Sheep Breeds. Animals : an open access journal from MDPI.

[CR14] Zeng B, Li S, Meng W, Zhang D (2019). An improved gray prediction model for China's beef consumption forecasting. PloS one.

